# Analysis of pilocytic astrocytoma by comparative genomic hybridization

**DOI:** 10.1054/bjoc.1999.1066

**Published:** 2000-02-21

**Authors:** D Sanoudou, O Tingby, M A Ferguson-Smith, V P Collins, N Coleman

**Affiliations:** Departments of Pathology, Clinical Veterinary Medicine, University of Cambridge, Cambridge, UK; Department of Histopathology, Addenbrooke's Hospital, Cambridge, UK; Institution of Oncology and Pathology, Karolinska Institute, Stockholm, S-17176, Sweden

**Keywords:** pilocytic astrocytoma, glioma, comparative genomic hybridization

## Abstract

Very little is known about genetic abnormalities involved in the development of pilocytic astrocytoma, the most frequently occurring brain tumour of childhood. We have analysed 48 pilocytic astrocytoma specimens using comparative genomic hybridization. Only five of 41 tumours from children showed abnormalities detectable by comparative genomic hybridization, and in each case this represented gain of a single chromosome. Interestingly, two of seven tumours from adults showed abnormalities, which were multiple and relatively complex. Six of the seven tumours showing abnormalities were from female patients (two adults and four children). The most frequently detectable abnormality was gain of 9q34.1-qter, which was present in three cases (two adult and one paediatric). © 2000 Cancer Research Campaign


*British Journal of Cancer* (2000) **82**(6), 1218–1222
© 2000 Cancer Research Campaign
Article no. bjoc.1999.1066, available online at http://www.idealibrary.com on IDEAL®

Short communication

### Analysis of pilocytic astrocytoma by comparative genomic hybridization

**D Sanoudou[1], O Tingby[3,4], MA Ferguson-Smith[2], VP Collins[1,3,4] and N Coleman[1,3]**

Departments of [1]Pathology and [2]Clinical Veterinary Medicine, University of Cambridge, Cambridge, UK; [3]Department of Histopathology, Addenbrooke's Hospital, Cambridge, UK; [4]Institution of Oncology and Pathology, Karolinska Institute, S-17176 Stockholm, Sweden

**Summary** Very little is known about genetic abnormalities involved in the development of pilocytic astrocytoma, the most frequently occurring brain tumour of childhood. We have analysed 48 pilocytic astrocytoma specimens using comparative genomic hybridization. Only five of 41 tumours from children showed abnormalities detectable by comparative genomic hybridization, and in each case this represented gain of a single chromosome. Interestingly, two of seven tumours from adults showed abnormalities, which were multiple and relatively complex. Six of the seven tumours showing abnormalities were from female patients (two adults and four children). The most frequently detectable abnormality was gain of 9q34.1-qter, which was present in three cases (two adult and one paediatric). © 2000 Cancer Research Campaign

**Keywords:** pilocytic astrocytoma; glioma; comparative genomic hybridization

Pilocytic astrocytoma (PA) is the most frequently occurring brain tumour in childhood. It is classified as grade I by the World Health Organization and does not tend to evolve into higher-grade tumours (Giannini and Scheithauer, 1997). Very little is currently known of the genetic abnormalities involved in the development of PA. More than 120 cases have been analysed cytogenetically, but no consistent abnormality has been identified. Of 119 paediatric cases examined, 38 showed detectable chromosomal abnormalities; eight adult cases have been examined and seven of these showed chromosomal abnormalities (Jenkins et al, 1989; Karnes et al, 1992; Ransom et al, 1992; Thiel et al, 1992; Ganju et al, 1994; Agamanolis and Malone, 1995; Debiec-Rychter et al, 1995; White et al, 1995; Bhattacharjee et al, 1997; Bigner et al, 1997; Zattara-Cannoni et al, 1998).

Only a small number of studies of molecular genetic abnormalities in PA has been undertaken to date. The cases included in these studies were either all paediatric or the patient age was not specified. Allelic loss has been reported on 17p, including at the *TP53* locus, although very few *TP53* mutations have been found (von Deimling et al, 1993; Lang et al, 1994; al-Sarraj et al, 1995; Phelan et al, 1995; Willert et al, 1995; Patt et al, 1996). Losses on 17q have also been found, and in some cases these encompassed the *NF1* locus (von Deimling et al, 1993; Platten et al, 1996). However, no *NF1* mutations were observed by single-strand conformation polymorphism analysis of 16 PA tumours (Scheurlen and Senf, 1995). A single case of PA was reported to have a non-sense mutation of *PTEN*, a gene that is frequently mutated in glioblastomas (Duerr et al, 1998). No other gene mutations have been reported and no loci have been shown to be consistently abnormal in PA.

*Received 7 July 1999*
*Revised 9 November 1999*
*Accepted 11 November 1999*

*Correspondence to*: N Coleman, Department of Histopathology, Addenbrooke's Hospital, Hills Road, Cambridge CB2 2QQ, UK

We have used comparative genomic hybridization (CGH) to screen for gross genomic copy number abnormalities in PAs from 48 patients. CGH enables the analysis of copy number imbalances across the genome in a single hybridization and avoids the need for cell culture (Kallioniemi et al, 1992).

### MATERIALS AND METHODS

### Tumour specimens

We studied archival frozen tumour tissue from patients operated at Karolinska Hospital Stockholm, Sahlgrenska Hospital Gothenburg and Addenbrooke's Hospital Cambridge. The clinical and histopathological data are summarized in Table 1. Of the 48 individual tumours studied, 41 were from patients less than 18 years of age and seven were from adults. We used the suffix 'a' to indicate tumour from primary operations and 'b' to indicate tumour from re-operations. Specimens PA8b, PA9b, PA45b, PA50b and PA53b were re-operated tumours for which tissue samples from the primary operation were not available.

### DNA extraction

High molecular weight DNA was isolated from the frozen tumour tissue pieces by homogenization in 4 M guadinium isothiocyanate buffer followed by ultracentrifugation on a caesium chloride (CsCl) gradient. The DNA was purified by proteinase K digestion and phenol–chloroform extraction. Reference genomic DNA was prepared from blood of one healthy male and one healthy female donor.

### Comparative genomic hybridization

CGH was carried out according to previously published protocols (Kallioniemi et al, 1992), with certain modifications. The reference and patient genomic DNA were differentially labelled

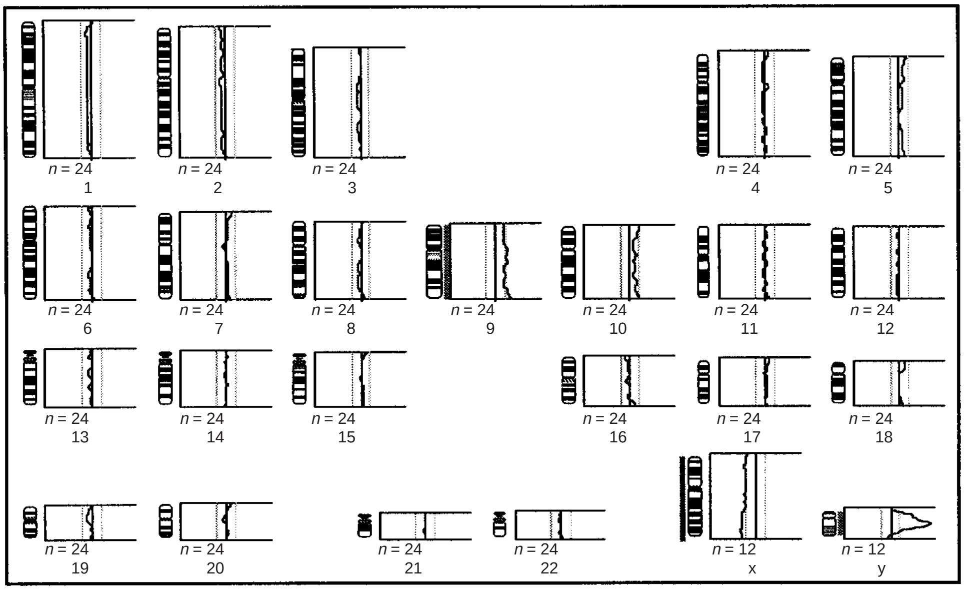

**Figure 1** CGH interpretation profile of case PA25a. The ration profile was determined from analysis of 12 separate metaphases. As the tumour arose in a male patient, we utilised control DNA from a female. There is evidence of gain of all of chromosome 9 in the tumour specimen.

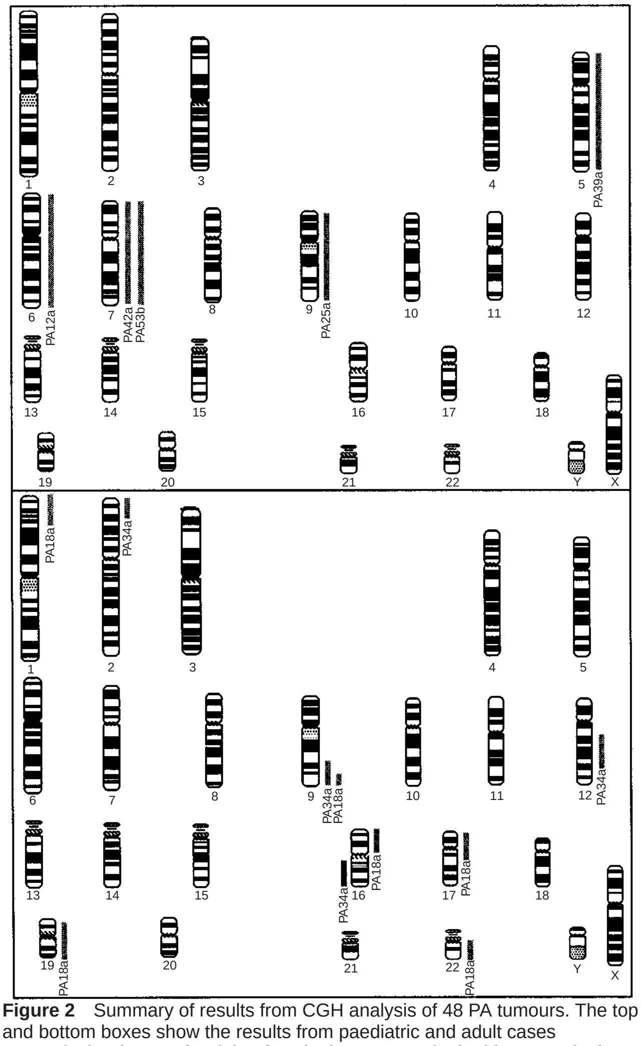

**Figure 2** Summary of results from CGH analysis of 48 PA tumours. The top and bottom boxes show the results from paediatric and adult cases respectively. Lines to the right of each chromosome in the ideogram depict regions of DNA gain, whilst lines to the left of each chromosome depict regions of DNA loss.

by DOP-PCR (Telenius et al, 1992) using biotin-16-dUTP and fluorescein isothiocyanate (FITC)-dUTP respectively. They were then co-hybridized onto normal human metaphases. The biotin label was detected with avidin-Cy3. Twelve to 15 metaphases were analysed for each specimen using the Vysis Quips CGH program. The thresholds used for the CGH ratio profiles were 1.2 for gain and 0.8 for loss. Each specimen was analysed at least twice.

### RESULTS

Seven of the 48 tumours showed chromosomal abnormalities detectable by CGH. A representative copy number karyogram is shown in Figure 1. In paediatric cases the abnormalities were gains and involved chromosomes 5, 6, 7, 9 (see Figure 2 for frequency). No loss of chromosomal regions was detected. Only five of the 41 paediatric tumours (12%) were abnormal. Of the 18 female and 23 male paediatric cases analysed, four female patients and only one male patient had detectable chromosomal abnormalities. In each of these only one aberration was seen and this involved the gain of a single chromosome. Two of the tumours gained a chromosome 7 while the other three gained a chromosome 5, a chromosome 6 or a chromosome 9.

Of the seven tumours from adult patients two (29%) showed multiple abnormalities, which included regional copy number changes as well as gains of whole chromosomes. Of the six female and one male adult cases only two female patients had detect-able aberrations. PA 18a had gains of 1p33-pter, 9q34.1-qter, 17q21.3-pter and whole gains of 19 and 22. PA 34a showed gains of 2p22-pter, 9q31-qter, 12q13.2-q23, together with loss of 16q.

There was no association between the presence of abnormalities detectable by CGH and the anatomical location of the tumours. The five abnormal paediatric cases were located in the posterior fossa (two cases), optic chiasm, left frontal lobe and hypothalamus (one case each), while the two abnormal adult cases were located

*© 2000 Cancer Research Campaign*

**Table 1** Clinical details relating to PA specimens analysed

| Case No | Age | Sex | %tum Tissue | Tumour location | CGH results |
|---|---|---|---|---|---|
| Paediatric cases with normal CGH results | | | | | |
| PA2a | 5 | M | 90 | Not known | normal |
| PA5a | 3 | F | 65 | cerebellum | normal |
| PA7a | 3 | F | 85 | Optic nerve | normal |
| PA8b | 8 | F | 75 | Cerebellum, ve | normal |
| PA9b | 13 | M | 85 | Hypothalamus | normal |
| PA14a | 5 | M | >90 | Posterior fossa | normal |
| PA15a | 14 | F | Not known | Vermis | normal |
| PA16a | 1 | M | 85 | Hypothalamus | normal |
| PA17a | 10 | F | 80 | Posterior fossa | normal |
| PA17b | 13 | F | 75 | Posterior fossa | normal |
| PA19a | 13 | M | 80 | 3rd ventricle | normal |
| PA20a | 6 | M | 90 | Posterior fossa | normal |
| PA23a | 5 | F | 90 | 4th ventricle | normal |
| PA24a | 18 | M | 90 | 4th ventricle | normal |
| PA28a | Not known | M | >80 | Right tempora | normal |
| PA28b | Not known | M | >85 | Not known | normal |
| PA29a | 15 | M | 90 | Posterior fossa | normal |
| PA30a | 16 | F | 85 | Right cerebella | normal |
| PA31a | 5 | F | 90 | Brainstem | normal |
| PA32a | 4 | M | Not known | 4th ventricle | normal |
| PA33a | 2 | M | 90 | 3rd ventricle | normal |
| PA37a | 16 | M | >85 | Posterior fossa | normal |
| PA40a | 5 | M | 85 | Optic nerve | normal |
| PA43a | 6 m | M | 90 | Hypothalmus | normal |
| PA44a | 1 | M | 85 | Optic nerve | normal |
| PA45b | 9 | M | >90 | Posterior fossa | normal |
| PA47a | 8 | F | 90 | Posterior fossa | normal |
| PA48a | 5 | F | 85 | Cerebellum | normal |
| PA49a | 3 | M | Not known | Vermis | normal |
| PA50b | Not known | M | 60–70 | 4th ventricle | normal |
| PA51a | 8 | M | Not known | Right cerebella | normal |
| PA52a | 6 | F | Not known | Posterior fossa | normal |
| PA54a | 6 | F | 100 | Cerebellum | normal |
| PA55a | 8 | F | 75 | Cerebellum | normal |
| PA56a | 18 | M | 80 | Posterior fossa | normal |
| PA57a | 10 | M | 80 | Posterior fossa | normal |
| PA57b | 13 | M | 100 | Posterior fossa | normal |
| PA58a | 10 | M | 90 | Not known | normal |
| PA60a | 7 | F | 80 | Posterior fossa | normal |
| Paediatric cases with abnormal CGH results | | | | | |
| PA12a | 17 | F | 85 | Posterior fossa | +6 |
| PA25a | 10 | M | 85 | Optic chiasm | +9 |
| PA39a | 11 | F | 90 | Posterior fossa | +5 |
| PA42a | 12 | F | 85 | Left frontal lobe | +7 |
| PA53b | 7 | F | 100 | Supra sellar | +7 |
| Adult cases with normal CGH results | | | | | |
| PA1a | 33 | F | 85 | Optic nerve | normal |
| PA3a | 32 | M | 80 | Cerebellum | normal |
| PA10a | 20 | F | 85 | Cerebellum | normal |
| PA35a | Not known | F | 80 | Midline | normal |
| PA41a | 23 | F | 85 | 4th ventricle | normal |
| Adult cases with abnormal CGH results | | | | | |
| PA18a | 33 | F | 85 | Pineal region | +1p33-pter, +9q34.1-qter, +16p, +17q21.3-pter, +19, +22 |
| PA34a | 48 | F | 90 | Vermis | +2p22-pter, +9q31-qter, +12q13.2-q23, −16q |

in the pineal region and posterior fossa. Two parts of specimen PA39a were analysed; interestingly, whereas one of these was normal, the other showed gain of chromosome 5. In all other tumours where two specimens were available no abnormalities were seen in the profiles of either tumour.

### DISCUSSION

The majority of cases analysed in this study show no chromosomal abnormality. Some aberrations, however, are detectable in a number of different chromosomes. The detectable chromosomal

 *© 2000 Cancer Research Campaign*

aberrations the paediatric tumours always involved gain of whole chromosomes. In adults gain of whole chromosomes or sub-chromosomal regions was seen, as well as regional loss. The difference in frequency and the types of aberration seen in the adult cases is interesting. A higher incidence of malignant progression has been reported in adult PAs compared to paediatric PAs (Giannini and Scheithauer, 1997).

The male to female ratio in our series was equal; there were 24 males and 24 females. However, four of the five paediatric tumours and both of the adult tumours with detectable abnormalities were from females. This finding is interesting and merits further investigation. There was no association between the presence of abnormalities detectable by CGH and the anatomical location of the tumours. The finding that one region of specimen PA39a was normal, whereas another showed gain of chromosome 5 is also of interest, and is consistent with the notion of clonal evolution in the neoplasm.

Our CGH results are consistent with data from previous cytogenetic studies. In the approximately 120 paediatric cases analysed cytogenetically to date, gains have been seen on chromosomes 5 (four cases), 6 (two cases), 7 (nine cases), 12 (two cases), 17 (two cases), 19 (three cases) and 22 (three cases) (Jenkins et al, 1989; Karnes et al, 1992; Agamanolis and Malone, 1995; Debiec-Rychter et al, 1995; White et al, 1995; Bhattacharjee et al, 1997; Bigner et al, 1997; Zattara-Cannoni et al, 1998). In the approximately eight adult cases analysed cytogenetically, gains have been seen for chromosomes 5 (two cases), 6 (three cases), 7 (two cases) and 12 (one case). Gain of chromosome 7 is one of the most common abnormalities in PA and has also been described as one of the most characteristic aberrations in glioblastoma multiforme (grade IV astrocytoma) (Liu et al, 1998). However, trisomy 7 has also been reported in non-neoplastic tissue (including brain tissue), both in vitro and in vivo, and the significance of its role in oncogenesis remains controversial (Johansson et al, 1993).

We detected several abnormalities that have not previously been reported in PA. These included gain of regions of chromosome 1p and chromosome 2p, in cases PA18a and PA34a respectively. Our most frequent finding (in three cases) was of gain on 9q, which was detected in both adult tumours with abnormalities and in one paediatric tumour (which showed gain of all of chromosome 9). The minimal region of gain is 9q34.1-qter. A variety of genes are located in this region including *ABL* (Abelson murine leukaemia), *VAV2* (Rho-family guanine-nucleotide exchange factor) and *PBX3* (pre B-cell leukaemia transcription factor).

In those cases of PA in which no abnormalities are detectable with CGH, it is not necessarily the case that no chromosomal aberrations are present. As CGH demonstrates loss or gain of DNA sequences, balanced translocations remain undetected. In addition, CGH will not detect polyploidy and will only detect DNA sequence copy number changes if they differ from the average copy number of chromosomes in the entire tumour specimen. It should be noted however that polyploidy in PA has been reported only in those rare cases which have undergone malignant transformation (Tomlinson et al, 1994; Mathew et al, 1996).

Abnormalities present in a small percentage of tumour cells may also not be detectable by CGH. In a recent cytogenetic study 12 out of 24 cases of paediatric PA were reported to be mosaic, and six of these cases showed random chromosomal gains and losses (Zattara-Cannoni et al, 1998). In addition, PA tumours may contain regions of DNA gain or loss that are beyond the resolution of CGH. With metaphase chromosome targets the technique can detect amplifications of approximately 2 MB (representing a product of amplicon size and copy number increase) and regions of DNA loss of 10 MB or greater (Kallioniemi et al, 1994).

In conclusion, we have demonstrated that major cytogenetic abnormalities are relatively rare in PA. Interestingly, however, the specimens from adults in our study showed more complex aberrations than those from paediatric patients. In the small number of abnormal cases no frequently occurring aberration is detectable, although there is some consistency between our findings and those from previous cytogenetic studies. Determination of mechanisms underlying the development of PA may require detailed genotypic analysis of tumour tissue, for example using developing DNA microarray technology.

### ACKNOWLEDGEMENTS

We would like to thank Mr Ian Roberts, Dr Fengtang Yang, Dr Charles Lee, Dr Willem Rens, Dr Cathy Boucher and Dr Carole Sargent for their advice and assistance during the course of this work. This work was supported by grants from the Joshua Gilbert Rhabdomyosarcoma Fund, Swedish Childhood Cancer Foundation, Karolinska Institute and Cancerfonden.

### REFERENCES

Agamanolis DP and Malone AM (1995) Chromosomal abnormalities in 47 pediatric brain tumors. *Cancer Genet Cytogenet* **81**: 125–134

al-Sarraj S, Bridges LR, Cawkwell L, Lewis FA and Quirke P (1995) p53 allelic imbalance in astrocytoma detected using fluorescent PCR of microsatellite repeat polymorphisms. *Neuropathol Appl Neurobiol* **21**: 344–351

Bhattacharjee MB, Armstrong DD, Vogel H and Cooley LD (1997) Cytogenetic analysis of 120 primary pediatric brain tumors and literature review. *Cancer Genet Cytogenet* **97**: 39–53

Bigner SH, McLendon RE, Fuchs H, McKeever PE and Friedman HS (1997) Chromosomal characteristics of childhood brain tumors. *Cancer Genet Cytogenet* **97**: 125–134

Debiec-Rychter M, Alwasiak J, Liberski PP, Nedoszytko B, Babinska M, Mrozek K, Imielinski B, Borowska-Lehman J and Limon J (1995) Accumulation of chromosomal changes in human glioma progression. A cytogenetic study of 50 cases. *Cancer Genet Cytogenet* **85**: 61–67

Duerr EM, Rollbrocker B, Hayashi Y, Peters N, Meyer-Puttlitz B, Louis DN, Schramm J, Wiestler OD, Parsons R, Eng C and von Deimling A (1998) PTEN mutations in gliomas and glioneuronal tumors. *Oncogene* **16**: 2259–2264

Ganju V, Jenkins RB, O'Fallon JR, Scheithauer BW, Ransom DT, Katzmann JA and Kimmel DW (1994) Prognostic factors in gliomas. A multivariate analysis of clinical, pathologic, flow cytometric, cytogenetic, and molecular markers. *Cancer* **74**: 920–927

Giannini C and Scheithauer BW (1997) Classification and grading of low-grade astrocytic tumors in children. *Brain Pathol* **7**: 785–798

Jenkins RB, Kimmel DW, Moertel CA, Schultz CG, Scheithauer BW, Kelly PJ and Dewald GW (1989) A cytogenetic study of 53 human gliomas. *Cancer Genet Cytogenet* **39**: 253–279

Johansson B, Heim S, Mandahl N, Mertens F and Mitelman F (1993) Trisomy 7 in nonneoplastic cells. *Genes Chromosomes Cancer* **6**: 199–205

Kallioniemi A, Kallioniemi OP, Sudar D, Rutovitz D, Gray JW, Waldman F and Pinkel D (1992) Comparative genomic hybridization for molecular cytogenetic analysis of solid tumors. *Science* **258**: 818–821

Kallioniemi OP, Kallioniemi A, Piper J, Isola J, Waldman FM, Gray JW and Pinkel D (1994) Optimizing comparative genomic hybridization for analysis of DNA sequence copy number changes in solid tumors. *Genes Chromosomes Cancer* **10**: 231–243

Karnes PS, Tran TN, Cui MY, Raffel C, Gilles FH, Barranger JA and Ying KL (1992) Cytogenetic analysis of 39 pediatric central nervous system tumors. *Cancer Genet Cytogenet* **59**: 12–19

*© 2000 Cancer Research Campaign*

Lang FF, Miller DC, Pisharody S, Koslow M and Newcomb EW (1994) High frequency of p53 protein accumulation without p53 gene mutation in human juvenile pilocytic, low grade and anaplastic astrocytomas. *Oncogene* **9**: 949–954

Liu L, Ichimura K, Pettersson EH and Collins VP (1998) Chromosome 7 rearrangements in glioblastoma; loci adjacent to EGFR are independently amplified. *J Neuropathol Exp Neurol* (in press)

Mathew P, Look T, Luo X, Ashmun R, Nash M, Gajjar A, Walter A, Kun L and Heideman RL (1996) DNA index of glial tumors in children. Correlation with tumor grade and prognosis. *Cancer* **78**: 881–886

Patt S, Gries H, Giraldo M, Cervos-Navarro J, Martin H, Janisch W and Brockmoller J (1996) p53 gene mutations in human astrocytic brain tumors including pilocytic astrocytomas. *Human Pathol* **27**: 586–589

Phelan CM, Liu L, Ruttledge MH, Muntzning K, Ridderheim PA and Collins VP (1995) Chromosome 17 abnormalities and lack of TP53 mutations in paediatric central nervous system tumours. *Hum Genet* **96**: 684–690

Platten M, Giordano MJ, Dirven CM, Gutmann DH and Louis DN (1996) Up-regulation of specific NF 1 gene transcripts in sporadic pilocytic astrocytomas. *Am J Pathol* **149**: 621–627

Ransom DT, Ritland SR, Kimmel DW, Moertel CA, Dahl RJ, Scheithauer BW, Kelly PJ and Jenkins RB (1992) Cytogenetic and loss of heterozygosity studies in ependymomas, pilocytic astrocytomas, and oligodendrogliomas. *Genes Chromosomes Cancer* **5**: 348–356

Scheurlen WG and Senf L (1995) Analysis of the gap-related domain of the neurofibromatosis type 1 (nf1) gene in childhood brain tumors. *Int J Cancer* **64**: 234–238

Telenius H, Pelmear AH, Tunnacliffe A, Carter NP, Behmel A, Ferguson-Smith MA, Nordenskjold M, Pfragner R and Ponder BA (1992) Cytogenetic analysis by chromosome painting using DOP-PCR amplified flow-sorted chromosomes. *Genes Chromosomes Cancer* **4**: 257–263

Thiel G, Losanowa T, Kintzel D, Nisch G, Martin H, Vorpahl K and Witkowski R (1992) Karyotypes in 90 human gliomas. *Cancer Genet Cytogenet* **58**: 109–120

Tomlinson FH, Scheithauer BW, Hayostek CJ, Parisi JE, Meyer FB, Shaw EG, Weiland TL, Katzmann JA and Jack CR, Jr. (1994) The significance of atypia and histologic malignancy in pilocytic astrocytoma of the cerebellum: a clinicopathologic and flow cytometric study. *J Child Neurol* **9**: 301–310

von Deimling A, Louis DN, Menon AG, von AK, Petersen I, Ellison D, Wiestler OD and Seizinger BR (1993) Deletions on the long arm of chromosome 17 in pilocytic astrocytoma. *Acta Neuropathol (Berl)* **86**: 81–85

White FV, Anthony DC, Yunis EJ, Tarbell NJ, Scott RM and Schofield DE (1995) Nonrandom chromosomal gains in pilocytic astrocytomas of childhood. *Hum Pathol* **26**: 979–986

Willert JR, Daneshvar L, Sheffield VC and Cogen PH (1995) Deletion of chromosome arm 17p DNA sequences in pediatric high-grade and juvenile pilocytic astrocytomas. *Genes Chromosomes Cancer* **12**: 165–172

Zattara-Cannoni H, Gambarelli D, Lena G, Dufour H, Choux M, Grisoli F and Vagner-Capodano AM (1998) Are juvenile pilocytic astrocytomas benign tumors? A cytogenetic study in 24 cases. *Cancer Genet Cytogenet* **104**: 157–160

 *© 2000 Cancer Research Campaign*

